# Inhibition of Endoplasmic Reticulum Stress by 4-Phenyl Butyric Acid Presents Therapeutic Effects on Periodontitis: Experimental Studies *In Vitro* and in Rats

**DOI:** 10.1155/2021/6618943

**Published:** 2021-03-03

**Authors:** Yang Feng, Rong Zhang, Yi-rong Wang, Fei Chen, Qiang Luo, Chuan Cai, Yang Jiao, Peng Xue

**Affiliations:** ^1^Institute of Stomatology, The First Medical Center, Chinese PLA General Hospital, Beijing 100853, China; ^2^Department of Traditional Chinese Medicine and Acupuncture, The Second Medical Centre, Chinese PLA General Hospital, National Clinical Research Center for Geriatric Diseases, Beijing 100853, China; ^3^Institute for the Prevention and Control of Major Health and Public Safety Events of Armed Police, No. 5 Fumin Street, Beijing 102600, China; ^4^State Key Laboratory of Military Stomatology & National Clinical Research Center for Oral Diseases & Shaanxi Key Laboratory of Oral Diseases, Department of Prosthodontics, School of Stomatology, The Fourth Military Medical University, Xi'an, Shaanxi Province 710032, China; ^5^Department of Stomatology, The 7th Medical Center, Chinese PLA General Hospital, Beijing 100700, China

## Abstract

This study investigated the probable mechanisms of endoplasmic reticulum (ER) stress involved in periodontitis *in vitro* and *in vivo*. We isolated periodontal ligament stem cells from periodontitis patients and healthy controls (P-PDLSCs and H-PDLSCs). To further simulate the periodontal microenvironment in patients, lipopolysaccharide (LPS) was used to treat H-PDLSCs. The results showed that periodontitis-related inflammation gave rise to the upregulated expression levels of ER stress representative genes including *GRP78*, *PERK*, *ATF4*, and *CHOP*. In contrast, the treatment of 4-phenyl butyric acid (4-PBA) remarkably suppressed ER stress and supported cell viability. The increased secretion of proinflammatory factors like TNF-*α*, IL-1*β*, and IL-6 and the activation of NF-*κ*B pathway were also attenuated by 4-PBA treatment. Moreover, 4-PBA treatment restored the impaired osteogenic differentiation ability of PDLSCs, as demonstrated by the upregulated expression levels of Runx2 and OCN as well as the enhanced Alizarin red staining. Local administration of 4-PBA could rescue alveolar bone resorption of LPS-induced periodontitis rats. Thus, our findings suggested ER stress might act as a promising therapeutic target against periodontitis.

## 1. Introduction

Periodontitis is among world's most prevalent inflammatory diseases, which has destructive effects on periodontal tissues including gingival, cementum, periodontal ligament, and alveolar bone [1]. It is reported that three quarters of the adult population worldwide have mild periodontal disease (gingivitis) at least and more than one-fifth present with severe and destructive periodontitis [2]. The clinical symptoms include gingival bleeding, formation of periodontal pocket, loss of connective tissue attachment, alveolar bone resorption, and eventually teeth exfoliation. The pathogenesis of periodontitis involves a local inflammatory reaction triggered by the presence of bacterial plaque [3]. Thus, the ultimate objective of periodontal treatment is not simply to control the inflammation, but more importantly, to recover the structure and feature of the damaged tissues in a diseased microenvironment.

Periodontal ligament stem cells (PDLSCs) are a group of heterogeneous mesenchymal stem cells that are located in the periodontal ligament [4]. It is widely accepted that the augmented aberrant differentiation and osteogenic differentiation dysfunction of PDLSCs are closely associated with the alveolar bone resorption during periodontitis [5, 6]. A complex network of signaling pathways governs the differentiation of PDLSCs in periodontitis. Generally, signaling pathways related to cell injury all are considered to be associated to the differentiation of PDLSCs, such as inflammation, autophagy-lysosome pathway, calcium homeostasis, ubiquitin-proteasome system, and endoplasmic reticulum (ER) stress. Generally, the inflammatory microenvironment composed of inflammatory cells and cytokines is noticeable to impede the osteogenic differentiation ability of PDLSCs. Nevertheless, anti-inflammatory therapy still has not achieved the goal to reserve aberrant differentiation of PDLSCs completely, implying there may be other regulatory mechanisms in periodontitis [7, 8]. Our previous studies demonstrated that unfolded protein accumulation in the ER activates the canonical unfolded protein response (UPR) representative genes including *GRP78*, *PERK*, *ATF4*, and *CHOP* and subsequently results in ER stress response, which may facilitate the aggravation of periodontitis [7, 9, 10]. Actually, numerous evidences have documented the intersections between ER stress and inflammation. For example, IRE*α*, a typical transmembrane receptor involved in UPR, could recruit TRAF2 to the ER membrane via the NF-*κ*B pathway to initiate cellular inflammatory responses [11]. However, there is still no available information to provide insight into a communicating network between ER stress and PDLSC fate determination especially osteogenic differentiation in periodontitis.

In the present study, we investigated the probable mechanisms of ER stress in periodontitis *in vitro* and *in vivo*. We found that 4-phenyl butyric acid (4-PBA) confers therapeutic effects on periodontitis by suppressing ER stress and inflammation and restoring PDLSC function. Our findings suggested that targeting ER stress might provide a prospective therapeutic strategy for periodontitis.

## 2. Materials and Methods

### 2.1. Cell Culture

We isolated periodontal ligament stem cells from periodontitis patients and healthy controls (P-PDLSCs and H-PDLSCs) as previously described [12, 13]. Briefly, periodontal ligament tissues were isolated from the middle third of the root surface and digested in 3 mg/ml collagenase I (Sigma-Aldrich, St Louis, MO, USA) for 2 h at 37°C. Periodontal ligament stem cells were cultured in 6-well culture dishes (Costar; Corning, NY, USA) in *α*-minimal essential medium (*α*-MEM; Gibco, Gaithersburg, MD, USA) supplemented with 0.292 mg/ml L-glutamine (Invitrogen, Carlsbad, CA, USA), 10% fetal bovine serum (FBS; Sijiqing, Zhejiang, China), 100 mg/ml streptomycin (Invitrogen), and 100 unit/ml penicillin (Invitrogen) in 5% CO_2_ at 37°C. Limiting dilution technique purified the stem cells into single cell colony cultures and then pooled and expanded. Cells from passages 2-5 were used in the following experiments. An identified ER stress inhibitor, 4-phenyl butyric acid (4-PBA, 5 mM; Sigma-Aldrich) was used to treat cells for 24 h, and lipopolysaccharide (LPS, 10 *μ*g/ml) was acquired from Pepro-Tech (Pepro-Tech, NJ, USA).

### 2.2. Real-Time Quantitative PCR

By using the SYBR Premix Ex Taq II kit (Takara, Shiga, Japan), RT-qPCR reactions were performed by using CFX96 Real-Time System (Bio-Rad, Hercules, CA, USA). We used standard settings: 94°C, 3 min; 94°C, 15 s; 60°C, 30 s; repeated 40 cycles, and then dissociation. Each assay ran in triplicate. The relative standard curve method calculated the arbitrary mRNA concentrations. The *^ΔΔ^*-*c*t method normalized to GAPDH. The primer sequences used in the present study are showed in Table 1.

### 2.3. Western Blot Analysis

Western blot analysis was performed as described in our previous studies [7, 9, 10]. RIPA lysis buffer extracted proteins, and then, G250 protein assay (Beyotime, Jiangsu, China) extracted soluble protein. Protein samples were loaded on 10% sodium dodecyl sulfate polyacrylamide gels (Invitrogen), transferred to polyvinylidene fluoride membranes (Bio-Rad), and blocked with 5% nonfat milk powder. Membranes were incubated overnight with the following primary antibodies for human Runx2 (Cell Signaling, Beverly, MA, USA), OCN (Santa Cruz, Dallas, TX, USA), NF-*κ*B (Santa Cruz), p-NF-*κ*B (Santa Cruz), and *β*-actin (Cowin Biotech, Beijing, China). Torseradish peroxidase- (HRP-) conjugated secondary antibody (Cowin Biotech) incubated the membranes for 2 h at room temperature. Protein signals were visualized by using the ECL Western Blotting Detection System (GE Healthcare, Piscataway, NJ, USA).

### 2.4. Enzyme-Linked Immunosorbent Assay (ELISA)

The samples were centrifuged at for 5 min 3000 g after a 15 min elution phase at room temperature in Eppendorf tube with 50 ml of PBS (Sigma-Aldrich). The supernatants were stored at -80°C until used. The concentrations of proinflammatory factors TNF-*α*, IL-1*β*, and IL-6 levels were assayed with commercial ELISA kits according to the manufacturer's instructions (BioSource, Camarillo, Calif., USA) in duplicate.

### 2.5. CCK-8 Assay

A total of 1 × 10^3^ cells of 0.1 ml *per* well were cultured in a 96-well plate in 10% FBS in three replicate wells. After drug treatment, 10 *μ*l of CCK-8 kit reagent (Beyotime) was added to 100 *μ*l of cell culture medium and incubated for 1.5 h at 37°C. The absorbance of each well at 450 nm was measured by using a microplate reader (Bio-Rad).

### 2.6. Osteogenic Differentiation In Vitro

The medium was changed to 2 mol/l *β*-glycerophosphate, 10 nmol/l dexamethasone, and 100 *μ*g/ml ascorbic acid (Sigma-Aldrich). RNA was extracted for 1 week for the osteogenic genes by using RT-qPCR analysis. Protein was extracted after 14 days for osteogenic markers by using Western blot analysis. Osteoblast calcium deposits were determined by staining with 1% Alizarin Red (Sigma-Aldrich) as previously described [14]. 1% cetylpyridinium chloride (Sigma-Aldrich) dissolved the mineralized nodules, and a spectrophotometer (Epoch, BioTek, USA) measured the OD value at 562 nm. Total protein quantity of each sample normalized the results to exclude the impact of cell numbers.

### 2.7. Drug Administration in Sprague–Dawley (SD) Rats of Experimental Periodontitis

A periodontitis model was established as described before [15]. We distributed three groups of nine eight-week-old SD rats: saline; LPS (10 *μ*g/day) and LPS (10 *μ*g/day) +4-PBA (5 nmol/day) with three rats *per* group. Then, the drug was injected between the first and second upper molars into the maxillary palatal gingiva in each group and repeated every two days for 7 days. The rats were anesthetized and euthanized by exsanguination. The SD rats' whole heads were removed, and a micro-CT system (Siemens Inveon Micro-CT, Munich, Germany) scanned and analyzed the maxillaries. The alveolar bone height of four different sites was recorded by measuring the length from the alveolar bone crest to the cemento-enamel junction (CEJ) in two molars. Body weight, heart/body weight, liver/body weight, spleen/body weight, adrenal gland/body weight, and kidney/body weight were measured to appraise the effect of drug administration on general condition.

### 2.8. Statistical Analysis

Data are represented as means ± standard deviations of each independent experiment (*n* = 3). Comparisons between the two groups were calculated by independent two-tailed unpaired Student's *t*-test and among numerous comparisons by the Bonferroni correction of analysis of variance in the GraphPad Prism software (San Diego, CA, USA). Significant differences were considered when *P* < 0 05.

## 3. Results

### 3.1. 4-PBA Reverses ER Stress in PDLSCs under Inflammatory Periodontitis Condition

**Figure 1:**
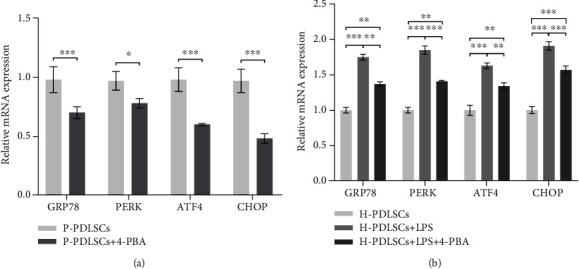
4-PBA reverses ER stress in PDLSCs under inflammatory periodontitis condition. RT-qPCR showed (a) the expression levels of UPR representative genes *GRP78*, *PERK*, *ATF4*, and *CHOP* in P-PDLSCs treated with or without 5 mM 4-PBA for 7 days; (b) the expression levels of UPR representative genes were measured in H-PDLSCs treated in the absence or presence of 10 mg/l LPS or LPS in association with 5 mM 4-PBA for 7 days. The consequences were normalized to the *GAPDH* gene, and data represent mean ± standard deviations (*n* = 3). ^∗^*P* < 0.05, ^∗∗^*P* < 0.01, ^∗∗∗^*P* < 0.001.

Our previous study has identified the upregulation of the classical UPR genes including *GRP78*, *PERK*, *ATF4*, and *CHOP* in primary P-PDLSCs [7]. To testify the effect of 4-PBA on ER stress in the inflammatory microenvironment, we treated P-PDLSCs with 4-PBA (5 mM) for 7 days. As shown in Figure 1(a), the UPR target genes in P-PDLSCs were significantly downregulated. To further simulate the periodontal microenvironment in patients, LPS was used to treat H-PDLSCs. The consequences showed that 4-PBA could significantly decrease the levels of URP target genes in LPS-treated H-PDLSCs. Nevertheless, their expression levels were still expressively higher than those in H-PDLSCs (Figure 1(b)).

### 3.2. 4-PBA Attenuates Inflammatory Response of P-PDLSCs and LPS-Treated H-PDLSCs

**Figure 2:**
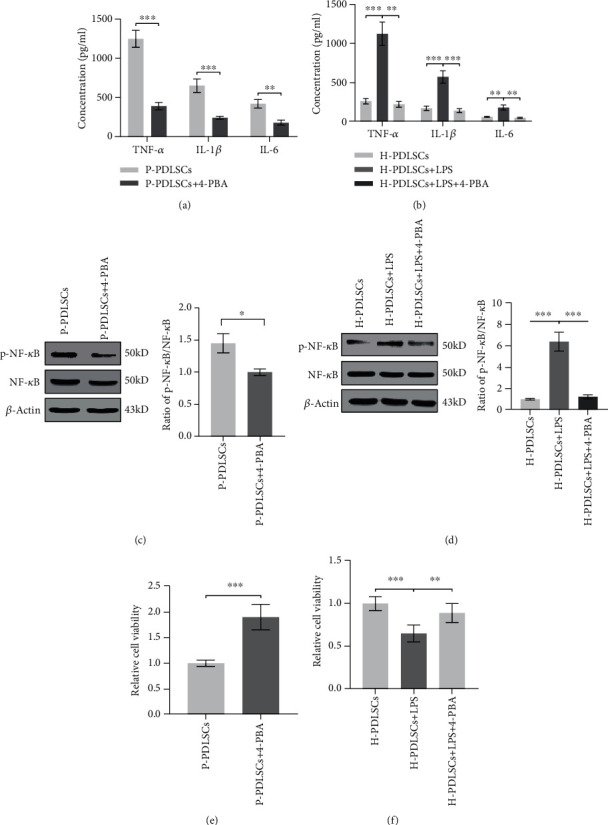
4-PBA attenuates inflammatory response of P-PDLSCs and LPS-treated H-PDLSCs. The secretion of proinflammatory factors TNF-*α*, IL-1*β*, and IL-6 was detected by using ELISA in (a) P-PDLSCs treated without or with 5 mM 4-PBA and (b) H-PDLSCs treated in the absence or presence of 10 mg/l LPS or LPS in association with 5 mM 4-PBA. Western blot analysis showed the expression levels of p-NF-*κ*B and NF-*κ*B, as well as the ratio of p-NF-*κ*B/NF-*κ*B in (c) P-PDLSCs treated without or with 5 mM 4-PBA or in (d) H-PDLSCs treated in the absence or presence of 10 mg/l LPS or LPS in association with 5 mM 4-PBA. Internal control used *β*-actin. (e, f) Cell viability was determined by using CCK-8 assay after two days treatment. The data represent mean ± standard deviations (*n* = 3). ^∗^*P* < 0.05, ^∗∗^*P* < 0.01, ^∗∗∗^*P* < 0.001.

Next, we assessed the expressions of proinflammatory cytokines in P-PDLSCs and LPS-treated H-PDLSCs. ELISA showed that treating with 4-PBA could significantly decrease the secretion of proinflammatory factors TNF-*α*, IL-1*β*, and IL-6 in P-PDLSCs and LPS-treated H-PDLSCs (Figures 2(a) and 2(b)). To explore the underlying mechanism, Western blot analysis was used to determine the expressions of p-NF-*κ*B and NF-*κ*B, and the results showed that treatment of 4-PBA could remarkably reduce the p-NF-*κ*B/NF-*κ*B ratio (Figures 2(c) and 2(d)). Further, the results of CCK-8 indicated that 4-PBA could support PDLSC viability in the inflammatory microenvironment (Figures 2(e) and 2(f)).

### 3.3. 4-PBA Restores the Impaired Osteogenic Differentiation Ability of PDLSCs in the Inflammatory Microenvironment

**Figure 3:**
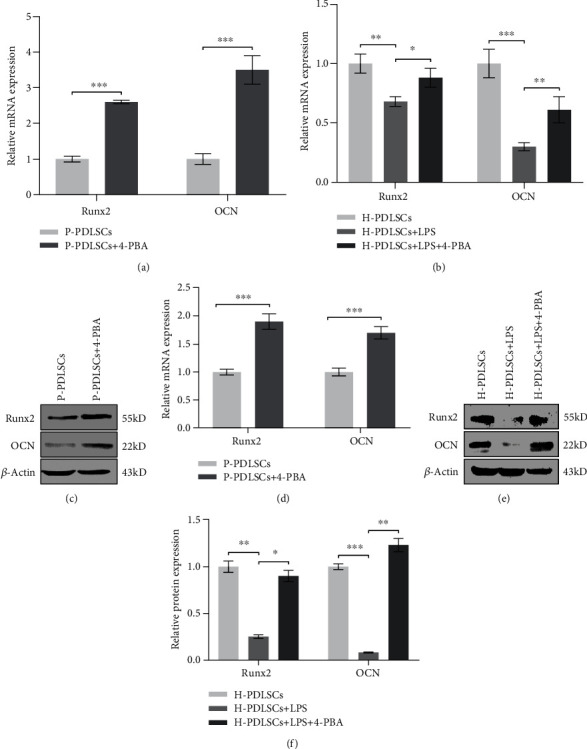
4-PBA restores the impaired osteogenic differentiation ability of PDLSCs in the inflammatory microenvironment. The mRNA and protein expression levels of Runx2 and OCN were determined by RT-qPCR after one week (a, b) and Western blot analysist after 14 days (c–f). P-PDLSCs were treated with or without 5 mM 4-PBA, and H-PDLSCs treated in the absence or presence of 10 mg/l LPS or LPS in association with 5 mM 4-PBA. The consequences were normalized to the *GAPDH* gene, and data represent mean ± standard deviations (*n* = 3). ^∗^*P* < 0.05, ^∗∗^*P* < 0.01, ^∗∗∗^*P* < 0.001.

**Figure 4:**
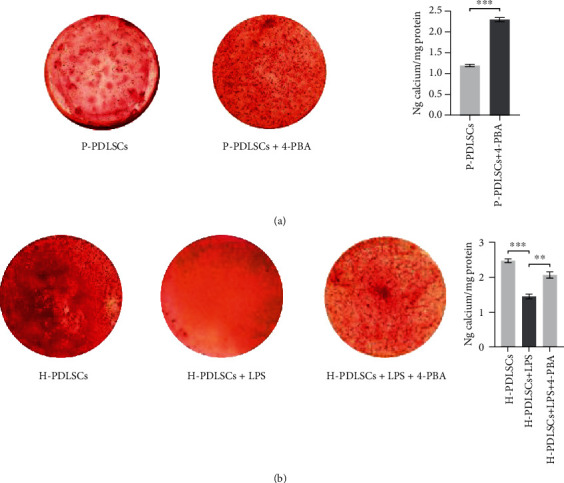
Alizarin red staining. (a) P-PDLSCs were treated without or with 5 mM 4-PBA, and (b) H-PDLSCs treated in the absence or presence of 10 mg/l LPS or LPS in association with 5 mM 4-PBA for 28 days. The data represent mean ± standard deviations (*n* = 3). ^∗^*P* < 0.05, ^∗∗^*P* < 0.01, ^∗∗∗^*P* < 0.001.

As shown in Figure 3, RT-qPCR (Figures 3(a) and 3(b)) and Western blot results (Figures 3(c)–3(f)) showed that the osteogenic differentiation associated biomarker proteins, like Runx2 and OCN, was significantly upregulated after 4-PBA treatment in both P-PDLSCs and LPS-treated H-PDLSCs. In addition, Alizarin red staining demonstrated that 4-PBA-treated cells formed more mineralization nodules in PDLSCs in the inflammatory microenvironment (Figures 4(a) and 4(b)).

### 3.4. Local Administration of 4-PBA Rescues Alveolar Bone Resorption in an LPS-Induced Periodontitis Rat Model

**Figure 5:**
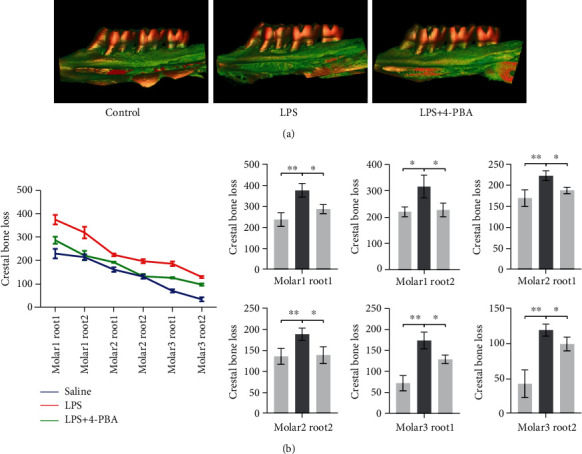
Local administration of 4-PBA rescues alveolar bone resorption in an LPS-induced periodontitis rat model. (a) Micro-CT acquired the representative images of the alveolar bone loss. (b) Alveolar bone resorption analysis of maxillary molars. Six sites for three molars were analyzed morphometrically (one site for each root of one tooth). The distance between the cement-enamel junction (CEJ) and alveolar crest of the mesial and distal roots of the three maxillary molars was assessed under a stereoscopic microscope. The results indicated more alveolar bone loss in the LPS (10 *μ*g/day) treated groups compared to the saline group (10 *μ*l). Local administration of 4-PBA (5 nmol/day) rescued the bone resorption induced by LPS. The data represent mean ± standard deviations (*n* = 3). ^∗^*P* < 0.05, ^∗∗^*P* < 0.01, ^∗∗∗^*P* < 0.001.

To further examine the effect of 4-PBA on periodontitis *in vivo*, LPS administration was recruited to establish a periodontitis rat model. As shown in Figure 5, the LPS group showed more excessive bone resorption as the farthest distance, from the alveolar bone crest to the CEJ, was observed in all the six sites. Whereas in the LPS+4-PBA group, the alveolar bone resorption of three molars was notably lessened, indicating that 4-PBA could rescue alveolar bone resorption in periodontitis. On the other hand, drug administration including LPS and LPS+4-PBA had little effect on the general condition of control rats (Table 2). Body weight, heart/body weight, spleen/body weight, adrenal gland/body weight, and kidney/body weight had little difference between the three groups, while liver/body weight was decreased in the LPS group compared to the control group and the LPS+4-PBA group (Table 2).

## 4. Discussion

In the present study, we provided a communicating network between ER stress and PDLSC function in periodontitis-related inflammation. To this end, P-PDLSCs and H-PDLSCs used as model cells and 4-PBA, an identified ER stress inhibitor, were used in this study. To simulate inflammatory periodontitis condition *in vitro* and *in vivo*, LPS was used to treat H-PDLSCs and also locally administrated in rats. The results showed that 4-PBA could downregulate the expression levels of ER stress-associated genes *GRP78*, *PERK*, *ATF4*, and *CHOP* in periodontitis-related inflammation. Besides, declined proinflammatory factors TNF-*α*, IL-1*β*, and IL-6 and suppression of NF-*κ*B signaling pathway after 4-PBA treatment indicated inhibited cellular inflammatory responses. Subsequently, upregulated Runx2 and OCN expression detected by RT-qPCR and Western blot analyses and enhanced Alizarin red staining suggested the restored osteogenic differentiation capability of PDLSCs in the inflammatory microenvironment. In addition, local administration of 4-PBA rescued alveolar bone resorption of LPS-induced periodontitis rats. Thus, our findings indicated 4-PBA exhibited a therapeutic potential against periodontitis both *in vitro* and *in vivo*.

Periodontitis is a characteristic inflammatory disease which is the main cause of periodontal tissue destruction and potentially tooth loss. Periodontal ligament stem cells are the core examinee stem cells for periodontal regeneration. Due to their self-renewal potential and multidifferentiation capability, PDLSCs have been widely used in tissue regeneration including periodontium [16]. The function and fate of PDLSCs are closely associated with the pathogenesis, progression, treatment, and prognosis in periodontitis [17, 18]. However, PDLSCs from periodontitis patients show deficient osteogenic differentiation ability in comparison with cells from healthy individuals [19]. Interestingly, osteogenic differentiation deficiency could not be retrieved after *ex vivo* culture and expansion [20]. In addition to chronic inflammation, the osteogenic capacity of PDLSCs might be affected by other factors. ER stress, caused by UPR, is an essential strategy in response to the drastic changes of the extracellular environment, which has been demonstrated to be involved in multiple oral diseases [7, 21, 22]. As previous studies reported, the IRE1*α* branch of the UPR might be associated with the secretion of proinflammatory cytokines in many kinds of cells such as endothelial cells and macrophages [23]. Endoplasmic reticulum stress impels macrophages to induce mature IL-1*β* in response to TLR4 stimulation via a TRIF- and caspase-8-dependent pathway [24]. In the present study, we found the upregulated expression levels of ER stress representative genes *GRP78*, *PERK*, *ATF4*, and *CHOP* both in P-PDLSCs and LPS-treated H-PDLSCs. These results indicated ER stress is a crucial pathogenesis in periodontitis. Moreover, periodontitis-related inflammation in the microenvironment resulted in lower cell viability, secretion of proinflammatory factors, and activation of NF-*κ*B pathway. In accordance with our previous studies [7, 21, 22], periodontitis impaired ER function and causes ER stress, which in turn results in deficient osteogenic differentiation of PDLSCs.

To investigate functional significance of ER stress in periodontitis, an identified ER stress inhibitor 4-PBA was used here. Previous studies have showed the therapeutic potential of 4-PBA for many kinds of human diseases [25–27]. Mechanically, 4-PBA could repress the overactivated ER stress and attenuate the UPR by stabilizing protein conformation, enhancing folding capacity of ER, and facilitating trafficking of mutant proteins [28–31]. In the present study, we found that the 4-PBA treatment remarkably suppressed ER stress and supported cell viability. The treatment of 4-PBA also reduced the enhanced secretion of proinflammatory cytokines by suppressing the activation of NF-*κ*B pathway. Indeed, previous studies have demonstrated that ER stress has been related to the activation of NF-*κ*B, a crucial transcription factor for many inflammatory processes [32]. We demonstrated that 4-PBA could interfere with the inflammation-related pathways in periodontitis, which might provide a new insight into the therapeutic mechanism of 4-PBA. Furthermore, 4-PBA treatment restored the impaired osteogenic differentiation ability of PDLSCs in the inflammatory microenvironment, and local administration of 4-PBA could rescue alveolar bone resorption of LPS-induced periodontitis rats. Our previous studies have shown that ER stress negatively affects the osteogenic differentiation function of PDLSCs via PERK and IRE1*α* pathways [7, 8]. Indeed, accumulating experimental evidence suggested the harnessing of ER stress has proven to be a particularly plausible therapeutic strategy for regulating bone metabolism and tissue repair, such as osteoarthritis [33], intervertebral disc degeneration [34], osteogenesis imperfecta [35, 36], and implant osseointegration [37]. It is noted that drug administration of LPS or LPS+4-PBA had adverse effects on the general condition of rats with lower liver/body weight than that of the control group, which may result from the hepatotoxicity of LPS [38].

Although our findings demonstrated that 4-PBA could suppress inflammation and restore the impaired osteogenic differentiation ability of PDLSCs by affecting the NF-*κ*B pathway, other molecular mechanisms have not been further studied. We also did not use multiple models of periodontitis, such as bacteria, ligature, or hypoxia, to validate these findings. These deficiencies will be explored in depth in future studies.

## 5. Conclusion

The present study investigated the possible mechanism ER stress associated with periodontitis. We investigated that inhibition of endoplasmic reticulum stress by 4-PBA suppressed ER stress and inflammation, supported cell viability, and restored the defective osteogenic differentiation of PDLSCs under inflammatory periodontitis conditions. Local administration of 4-PBA rescued alveolar bone resorption in an LPS-induced periodontitis rat model. Our findings suggested the potential of harnessing ER stress in order to develop a novel therapeutic approach for periodontitis.

## Figures and Tables

**Table 1:** Primer sequence used for RT-qPCR.

Gene	Primer	Sequence 5′ to 3′
*PERK*	Forward	GTGATAAAGGTTTCGGTTGCTG
	Reverse	TGTTTTCTGTGGCTCCTCTGG
*GRP78*	Forward	TCAAGTTCTTGCCGTTCAAGG
	Reverse	AAATAAGCCTCAGCGGTTTCTT
*ATF4*	Forward	CATGGGTTCTCCAGCGACA
	Reverse	TCTGGCATGGTTTCCAGGTC
*CHOP*	Forward	CAAGAGGTCCTGTCTTCAGATGA
	Reverse	TCTGTTTCCGTTTCCTGGTTC
*Runx2*	Forward	CCCGTGGCCTTCAAGGT
	Reverse	CGTTACCCGCCATGACAGTA
*OCN*	Forward	CCCAGGCGCTACCTGTATCAA
	Reverse	GGTCAGCCAACTCGTCACAGTC
*GAPDH*	Forward	CTGCAAGAACAGCATTGCAT
	Reverse	GACCACCTGGTCCTCAGTGT

**Table 2:** Effect of 4-PBA local administration on rats with experimental periodontitis. The body weight, heart/body weight, liver/body weight, spleen/body weight, adrenal gland/body weight, and kidney/body weight were analyzed in the saline group, LPS group, and LPS in combination with the 4-PBA group. The data represent mean ± standard deviations (*n* = 3). ^∗^*P* < 0.05.

	Saline (×10^−3^kg)	LPS (×10^−3^kg)	LPS+4PBA (×10^−3^kg)
Body weight	0.36 ± 0.2	0.32 ± 0.1	0.33 ± 0.05
Heart/body weight	3.08 ± 0.52	3.38 ± 0.31	2.67 ± 0.36
Liver/body weight	47.37 ± 3.31	34.09 ± 2.65^∗^	39.33 ± 7.3
Spleen/body weight	2.69 ± 0.49	2.47 ± 0.13	2.6 ± 0.82
Adrenal gland/body weight	0.21 ± 0.08	0.20 ± 0.1	0.26 ± 0.06
Kidney/body weight	8.10 ± 0.29	7.98 ± 0.86	7.87 ± 1.31

## Data Availability

All data generated or analyzed during this study are included in this published article.
